# Genetic variation of St. Louis encephalitis virus

**DOI:** 10.1099/vir.0.2008/000190-0

**Published:** 2008-08

**Authors:** Fiona J. May, Li Li, Shuliu Zhang, Hilda Guzman, David W. C. Beasley, Robert B. Tesh, Stephen Higgs, Pushker Raj, Rudy Bueno, Yvonne Randle, Laura Chandler, Alan D. T. Barrett

**Affiliations:** 1 Center for Biodefense and Emerging Infectious Diseases, University of Texas Medical Branch, Galveston, TX 77555-0609, USA; 2 Sealy Center for Vaccine Development, University of Texas Medical Branch, Galveston, TX 77555-0609, USA; 3 Institute for Human Infections and Immunity, University of Texas Medical Branch, Galveston, TX 77555-0609, USA; 4 Department of Pathology, University of Texas Medical Branch, Galveston, TX 77555-0609, USA; 5 Department of Microbiology and Immunology, University of Texas Medical Branch, Galveston, TX 77555-0609, USA; 6 Texas Department of State Health Services, Austin, TX, USA; 7 Harris County Public Health and Environmental Services, Mosquito Control Division, 3330 Old Spanish Trail, Houston, TX 77021, USA; 8 Laboratory Medicine, Philadelphia VA Medical Center, 3900 Woodland Avenue, Philadelphia, PA 19104, USA

## Abstract

St. Louis encephalitis virus (SLEV) has been regularly isolated throughout the Americas since 1933. Previous phylogenetic studies involving 62 isolates have defined seven major lineages (I–VII), further divided into 14 clades. In this study, 28 strains isolated in Texas in 1991 and 2001–2003, and three older, previously unsequenced strains from Jamaica and California were sequenced over the envelope protein gene. The inclusion of these new sequences, and others published since 2001, has allowed better delineation of the previously published SLEV lineages, in particular the clades of lineage II. Phylogenetic analysis of 106 isolates identified 13 clades. All 1991 and 2001–2003 isolates from Nueces, Jefferson and Harris Counties (Texas Gulf Coast) group in clade IIB with other isolates from these counties isolated during the 1980s and 1990s. This lack of evidence for introduction of novel strains into the Texas Gulf Coast over a long period of time is consistent with overwintering of SLEV in this region. Two El Paso isolates, both from 2002, group in clade VA with recent Californian isolates from 1998–2001 and some South American strains with a broad temporal range. Overall, these data are consistent with multiple introductions of SLEV from South America into North America, and provide support for the hypothesis that in most situations, SLEV circulates within a locality, with occasional incursions from other areas. Finally, SLEV has much lower nucleotide (10.1 %) and amino acid variation (2.8 %) than other members of the Japanese encephalitis virus complex (maximum variation 24.6 % nucleotide and 11.8 % amino acid).

## INTRODUCTION

St. Louis encephalitis virus (SLEV) is a member of the family *Flaviviridae*, the genus *Flavivirus*, and is classified within the Japanese encephalitis virus (JEV) complex along with other important pathogens such as JEV, Murray Valley encephalitis virus (MVEV) and West Nile virus (WNV) (Thiel *et al.*, 2005). SLEV has been detected in, and in many cases isolated from, mosquitoes, birds and mammals throughout North, Central and South America, from southern Canada to Argentina (Reisen, 2003). Epidemics of SLEV infection occur sporadically, with large outbreaks often being preceded by smaller outbreaks in previous years, and are generally associated with increasing numbers of infected mosquitoes and birds (Day & Stark, 1999; Day, 2001). Since it was first identified during the 1933 outbreak in St Louis, Missouri, there have been a number of epidemics of encephalitis attributed to SLEV, resulting in more than 1000 deaths, more than 10 000 cases of severe illness, and more than 1 000 000 mild or subclinical infections (Reisen, 2003). Fatality rates increase with age, with those below 49 years old exhibiting only 5 % mortality, while those above 70 years of age show 23 % mortality (Day, 2001).

SLEV utilizes different mosquito hosts in different regions. In the northern half of the USA, SLEV is most often isolated from *Culex pipiens*; while in Florida, it is most often isolated from *Culex nigripalpus*. In the south-west, it is most often isolated from *Culex quinquefasciatus*; but it is transmitted by *Culex tarsalis* in rural areas (reviewed by Day, 2001). A number of studies have provided evidence that, rather than being reintroduced annually, SLEV circulates within a region from season to season (Chandler *et al.*, 2001; Kramer & Chandler, 2001), although the method of overwintering has yet to be clearly determined. SLEV has been shown to persist in *Culex* species for more than a month, and isolates have been obtained from overwintering *C. tarsalis* and *C. pipiens* mosquitoes (Bailey *et al.*, 1978; Reeves *et al.*, 1958), suggesting that this is the most likely method of season-to-season persistence. However, experimental vertical transmission of SLEV in mosquitoes has also been reported (Francy *et al.*, 1981; Hardy *et al.*, 1980, 1984; Nayar *et al.*, 1986).

The SLEV genome, like those of other flaviviruses, is approximately 11 kb, single-stranded, positive-sense RNA and encodes a polyprotein flanked by 5′ and 3′ untranslated regions. The polyprotein is co- and post-translationally processed into the three structural proteins, capsid (C), premembrane/membrane (prM/M) and envelope (E) and seven non-structural proteins: NS1, NS2A, NS2B, NS3, NS4A, NS4B and NS5. Previous phylogenetic studies based on the E gene have shown the existence of seven lineages, grouping most viruses geographically (Kramer & Chandler, 2001). However, there is some evidence to suggest spread of individual variants across the USA, as in the case of the 1975 epidemic, with isolates from Tennessee, Mississippi and California grouping together (Kramer & Chandler, 2001) and variation of genotype within a single geographical location (Kramer *et al.*, 1997; Reisen *et al.*, 2002). There is no evidence of correlation between phenotypic characteristics such as virulence and phylogenetic groupings (Kramer *et al.*, 1997; Trent *et al.*, 1980). Since the introduction of WNV into southern California, no SLEV isolates have been detected despite substantial surveillance of mosquito populations (Fang & Reisen, 2006), suggesting that these viruses may not be able to coexist when sharing the same mosquito vector. Likewise, very few isolates of SLEV have been obtained in East Texas since the introduction of WNV into that region in 2002. Previously, the only examples of closely related viruses co-circulating simultaneously in the same mosquito population occurred in Australia with Kunjin virus (a subtype of WNV), JEV and MVEV occurring endemically in the same ecological niche (Hall *et al.*, 2002; Johansen *et al.*, 2000; Kay *et al.*, 1984), and in India where WNV and JEV circulate together (Carey *et al.*, 1968a, b).

SLEV was first detected in Harris County (Houston), Texas, during the countrywide epidemic of 1964. During this epidemic, more cases were reported in Harris County than in any other county in the USA (Luby *et al.*, 1967), and indeed, this trend has continued with Harris County often reporting more SLEV isolations than any other county in the USA (Tsai *et al.*, 1988; http://www.cdc.gov/ncidod/dvbid/sle/). The current study investigated the genetic relationships of a number of recent isolates from Texas, some older isolates from California and one from Jamaica, with previously studied isolates and proposes an updated classification of some SLEV clades. Furthermore, amino acid variation in the E protein was found to be very limited compared with variation between strains of other viruses in the JE complex.

## METHODS

### Viruses.

The viruses used in these studies are described in Table 1 and were obtained from the Texas Department of State Health Services in Austin, Harris County Mosquito Control Division, Houston, or the World Reference Center for Emerging Viruses and Arboviruses at the University of Texas Medical Branch at Galveston. Viruses were passaged once in Vero cells grown in minimal essential medium (Gibco) supplemented with 100 U penicillin ml^−1^, 100 μg streptomycin ml^−1^, 0.1 mM essential amino acids, 1 mM sodium pyruvate and 2 % bovine growth serum. Aliquots of virus were stored at −80 °C.

### RNA isolation, RT-PCR and sequencing.

RNA was isolated from supernatants harvested from infected cells when cytopathic effect was evident by using the QIAamp viral RNA extraction kit (Qiagen) and following the manufacturer's instructions. Viral RNA was amplified by RT-PCR using the Titan RT-PCR kit (Roche) and primers F880 and B2581 or B2586 (see Kramer & Chandler, 2001 for primer sequences and PCR conditions). PCR products were sequenced directly by standard methods with F880, B2581/B2586, SLE1 (5′-GTGCATGGTTCAACGGACTC-3′) and SLE2 (5′-GGTCACAGAGATGGGAACCC-3′) primers at the Protein Chemistry Core laboratory at the University of Texas Medical Branch at Galveston, or cloned into pGEM-T Easy (Promega) before sequencing.

### Phylogenetic analysis.

Sequences were analysed using ContigExpress and AlignX from the Vector NTI suite (Invitrogen). Neighbour-joining, parsimony and maximum-likelihood phylogenetic trees were constructed using the phylip package (Felsenstein, 1989). All trees were rooted using the E gene sequences of JEV (strain Ling; GenBank accession no. L78128), MVEV (strain 1-51; GenBank accession no. NC_000943) and WNV (strain NY99; GenBank accession no. DQ211652). Recombination was analysed using the Recombination Detection Program (Martin *et al.*, 2005).

## RESULTS

### Isolates analysed in this study

Since the 2001 publication by Kramer & Chandler (2001) that analysed 62 isolates from the Americas, a number of new isolates and some older isolates of SLEV have been sequenced. Published SLEV sequences since 2001 include 12 isolates from the Coachella Valley in California (Reisen *et al.*, 2002), two 2005 isolates from Argentina (Diaz *et al.*, 2006) and one 2004 human isolate from Brazil (Santos *et al.*, 2006). During our study, we have determined the E gene sequence of 25 isolates from Texas, one from Jamaica and two from California.

### Phylogenetic analysis of 106 isolates of SLEV

**Fig. 1. f1:**
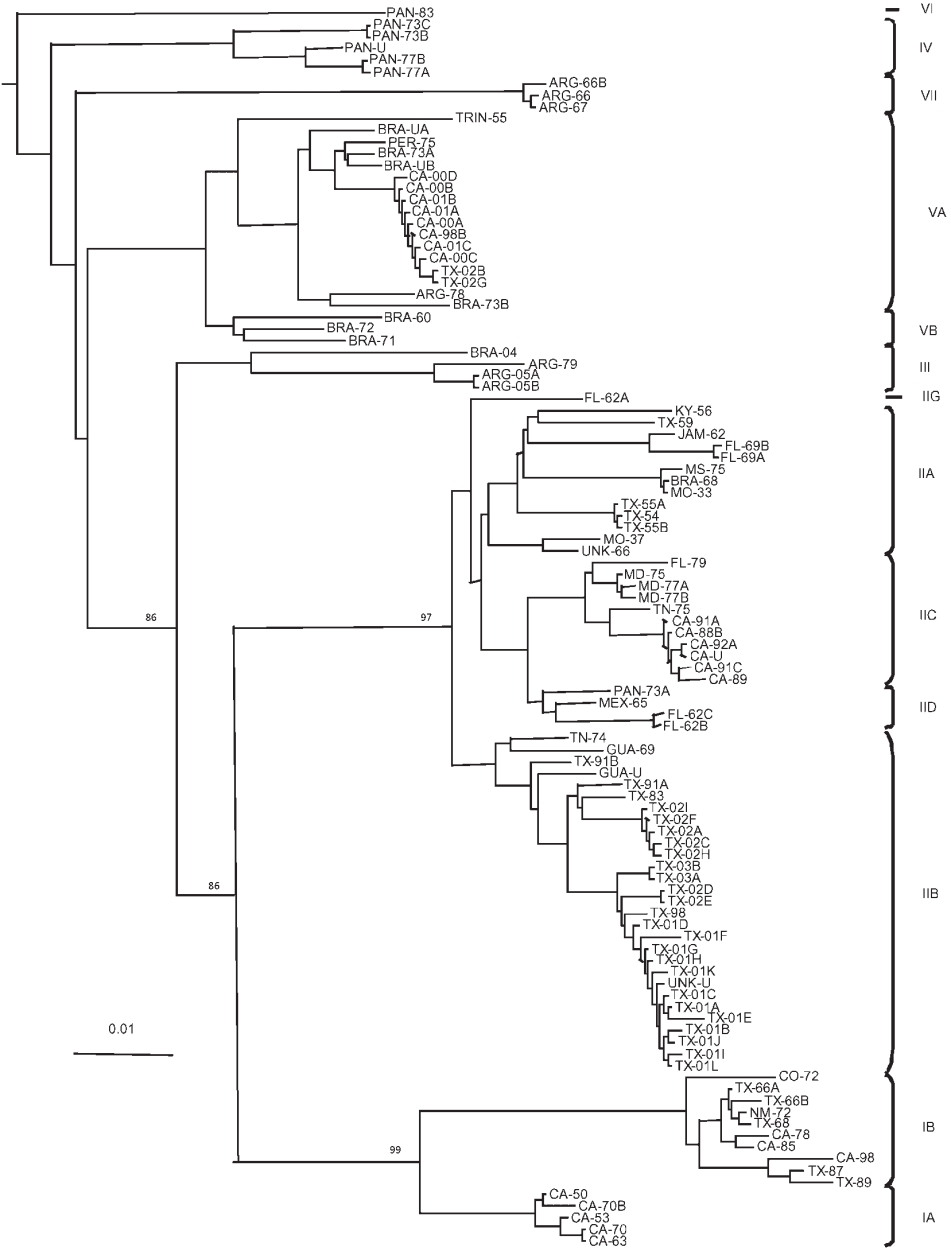
Neighbour-joining tree of the E gene of all available SLEV isolates. Numbers at nodes are percent bootstrap (of 100 replicates). Isolates are named according to location and year of isolation. CA, California; TX, Texas; NM, New Mexico; CO, Colorado; MS, Mississippi; KY, Kentucky; MO, Missouri; TN, Tennessee; MD, Maryland; FL, Florida; JAM, Jamaica; MEX, Mexico; BRA, Brazil; ARG, Argentina; GUA, Guatemala; TRIN, Trinidad; PAN, Panama; PER, Peru; UNK, unknown USA location. See Table 1 and Supplementary Table S1 for strain designations. The tree was rooted with JEV (GenBank accession no. L78128), MVEV (GenBank accession no. NC_000943) and WNV (GenBank accession no. DQ211652), but these have been removed to allow better visualization of branch lengths.

Phylogenetic analysis of the E protein gene sequences of all available (106) SLEV isolates confirmed the classification of SLEV into seven major lineages as proposed by Kramer & Chandler (2001), but indicated that further refinement of the clades within these lineages was justified. Several different methods for the construction of phylogenetic trees were performed, including neighbour joining (see Fig. 1), parsimony and maximum likelihood (data not shown). All of these methods confirmed the same seven lineages (I–VII); however, there was some variation in the exact order of branching within each lineage. A total of 13 clades were identified (IA, IB, IIA, IIB, IIC, IID, IIG, III, IV, VA, VB, VI and VII). All SLEV isolates exhibited high levels of identity (above 89.9 %), with identity within lineages even higher (>94.5 %), resulting in inconsistencies in minor branching when different tree construction methods were used. However, most bootstrap values for major branches were high (Fig. 1), showing that division of SLEV into the seven major lineages is robust.

### Re-evaluation of some SLEV nucleotide sequences

Recent sequencing of full-length SLEV genomes (GenBank accession nos EF158048–EF158070, G. J. Baillie, E. Waltari & S. L. Perkins, unpublished) has revealed some discrepancies between the previously published E sequences of two SLEV strains: Parton [MO-33; GenBank accession nos AF205509 and EF158070, see Table 1 and Supplementary Table S1 (available in JGV Online) for isolate details] and GMO-94 (GUA-69; GenBank accession nos AF205513 and EF158051). Our sequence of MO-33 is identical to that of the Brazilian isolate, SpAn9398 (BRA-68) (Kramer & Chandler, 2001). The MO-33 strain E gene was resequenced in this study by cloning the PCR product into a shuttle plasmid (pGEM-T Easy) and sequencing four representative clones. These clones were all identical to the published sequence of BRA-68. Unfortunately, BRA-68 was not available for use in this study. It has been proposed that GUA-69 resulted from a recombination between SLEV strains CorAn9124 (ARG-66; GenBank accession no. AF289617) and TNM4-711K (TN-74; GenBank accession no. AF205501) (Twiddy & Holmes, 2003). Our sequence for GUA-69 is similar to TN-74 (98.7 % identity) with no evidence of the virus being a recombinant (data not shown).

### SLEV isolates from California are found in multiple lineages

Two of the newly sequenced strains, BFN1324 (CA-70B) and BFS508 (CA-50), fall within clade IA. Of the other previously sequenced Californian isolates, those isolated before 1970 occur in this clade, while subsequent isolates from 1985 to 1998 fall within clade IB along with isolates from Texas and New Mexico, or clade IIC along with isolates from Tennessee and Maryland (Kramer & Chandler, 2001), while the recent isolates, from 1998 to 2001 (Reisen *et al.*, 2002), fall in clade VA.

### A 1962 isolate from Jamaica groups with older, widespread USA isolates

The Jamaican isolate sequenced in this study, J7532 (JAM-62), groups with isolates from the USA in clade IIA. This clade, which now appears to be extinct, includes the original SLEV isolate from the 1933 outbreak in Missouri (MO-33), along with other pre-1969 isolates from across the USA.

### Phylogenetic distribution of Texas isolates

Most recent isolates from Texas were obtained from the Gulf Coast counties (Jefferson, Harris and Nueces) in 1991 and 2001–2003. Sequences of these isolates showed a very low level of nucleotide divergence (between 98.1 and 100 % identity), and indeed, most of the changes are non-coding, with many of these isolates having identical amino acid sequences. All isolates grouped in clade IIB with other Texas Gulf Coast isolates from 1983 [83V4953 (TX-83)], 1991 [PV1-2419 (TX-91)] and 1998 [98V3181 (TX-98)], a 1974 Tennessee isolate (TN-74) and two Guatemalan strains [GUA-69 and 78A28 (GUA-U)]. Thus, only clade IIB contains isolates from these Texas Gulf Coast counties. Pre-2002 isolates from northern counties in Texas, such as Dallas, Hale and El Paso counties, are in clade IB, while two 2002 isolates from El Paso (sequenced in this study) are in clade VA along with older isolates from Peru, Argentina and Brazil and recent (1998–2001) isolates from the Coachella Valley in California. Both El Paso isolates are identical and show between 99.7 and 100 % identity with 1998–2001 isolates from the Coachella Valley in California, but only 91.8–92.4 % identity with the 2001–2002 Gulf Coast Texas isolates. A 1987 El Paso isolate [PV7-3389 (TX-87)] has only 91.8 % identity with the new isolates. TX-87 shares greater identity with isolates from Dallas and Hale County (north Texas) from 1966, 1968 and 1989 than with any other Texan isolate. A number of strains from unknown locations in Texas isolated in the 1950s and an isolate from the same era from the Rio Grande Valley are in clade IIA.

### Variation in the E protein amino acid sequence

**Fig. 2. f2:**
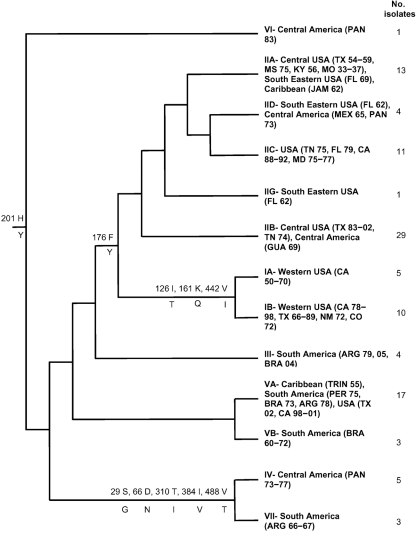
Simplified tree summarizing the phylogenetic relationships of the SLEV lineages, geographical location and number of isolates in each lineage and the amino acid changes associated with specific lineages.

**Fig. 3. f3:**
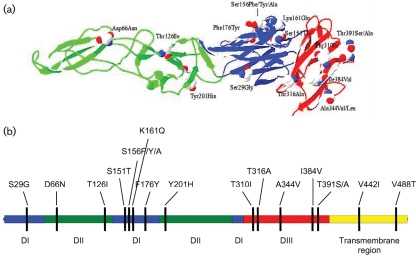
Position of amino acid changes on the envelope. Domain I is coloured blue, domain II is coloured green, domain III is coloured red and the transmembrane domain is coloured yellow. (a) Three dimensional location of changes using the WNV E structure (PDB 2HG0), side view. The bottom surface of the protein interacts with the virion. (b) Position on the linear E protein.

Comparison of the 501 aa in the E protein of all the available sequenced SLEV strains reveals a limited number of amino acid substitutions across all the sequences. A number of changes are specific for individual lineages, as shown in Table 2, and some define specific nodes in the phylogenetic tree (Fig. 2). The positions of the amino acid changes specific for different lineages are shown on the WNV E protein structure in Fig. 3. Although these changes occurred across all three domains in the E protein ectodomain, they clustered on the surface of the protein in domains I and III. None of these changes correspond with any previously published virulence factors, none appear to be in functionally important regions and there is no evidence of antigenic variation.

There was no apparent relationship between glycosylation status and lineage. SLEV has previously been shown to encode two potential glycosylation sites in the E protein (Trent *et al.*, 1987; Vorndam *et al.*, 1993). Although some strains are non-glycosylated, either by mutation of the glycosylation motif or by not utilizing the coded sites (Vorndam *et al.*, 1993), the site at position 154 is conserved in most other flaviviruses, with the exception of some strains of yellow fever virus (Ballinger-Crabtree & Miller, 1990; Schlesinger *et al.*, 1983), WNV (Adams *et al.*, 1995; Wengler *et al.*, 1985; Wright, 1982) and Alfuy virus (May *et al.*, 2006), and is most likely to be the position that is glycosylated. Of the 106 SLEV sequences used in this study, only 14 do not code for this glycosylation site (Ser to Phe or Tyr at position 156), while 45 isolates do not code for the second potential glycosylation site at position 314 (Thr to Ala at position 316).

## DISCUSSION

The current study aimed to expand the phylogenetic analysis of SLEV by Kramer & Chandler (2001) by incorporating isolates sequenced since 2001, focusing in particular on new isolates from Texas. Twenty-eight new isolates and 78 sequences from GenBank were included in this study; in total 106 E gene sequences were analysed. Of the 28 new isolates, 25 were from Texas, including isolates from 1991 and 2001–2003. Interestingly, no isolates of SLEV were obtained in Harris County in 2004 or 2006, and only one isolate in 2005 (R. B. Tesh, unpublished data). Although we lack statistical evidence to draw specific conclusions regarding the lack of SLEV isolates in those years, it is interesting to note that the paucity of SLEV isolates has been seen following the introduction of WNV into this area. Furthermore, when WNV arrived in Texas in 2002, mosquito pools were found to have both SLEV and WNV viruses (Lillibridge *et al.*, 2004), while this has not been observed subsequently (R. B. Tesh, unpublished data). This is consistent with the situation in California (Fang & Reisen, 2006). This situation may, in part, be explained by the relatively high prevalence of WNV antibodies in birds in regions with circulation of both viruses, a condition which inhibits SLEV virus replication in WNV-immune birds (Fang & Reisen, 2006). Surveillance of SLEV isolates in all areas of the USA over the next few years may determine if WNV is indeed replacing SLEV.

The geographical range of SLEV isolates in each clade is shown in Fig. 2. Kramer & Chandler (2001) described the division of SLEV isolates into seven genetic lineages (I–VII), mostly corresponding to their geographical location. The isolates newly sequenced in this study were distributed among lineages I, II and V, and the inclusion of other sequences published since 2001 has also allowed lineage III, previously containing only one isolate from Argentina, to be further defined.

Lineages IV, VI and VII contain South American isolates only and are basal to the other lineages in the tree, suggesting that these lineages are older. Lineage V is a combination of Central and South American strains, plus North American strains from California and West Texas, while lineages I and II are composed of isolates from North America only, suggesting that SLEV originated in South America and has subsequently spread into North America, probably on multiple occasions. Lineage I is divided into two clades containing isolates from the western parts of the USA, with clade IA composed of three Californian isolates from 1953, 1963 and 1970 and clade IB composed of other, more recent (1978 and 1983) isolates from California, older viruses from New Mexico and Colorado, and viruses from west and north Texas isolated between 1966 and 1989. The two newly sequenced isolates from California, CA-70B and CA-50, isolated in 1970 and 1950, respectively, fall into clade IA along with the three other Californian isolates from the same era. All isolates from California after the late 1980s are in lineage II, suggesting that the isolates circulating in California after this time were introduced from South America, and may have replaced the previously circulating isolates.

Lineage II is heterogeneous and contains isolates from throughout the USA. This lineage has 58 isolates and was divided by Kramer & Chandler (2001) into six major clades (A–F). The addition of the new isolates sequenced in this study has allowed greater differentiation of the clades in this lineage, resulting in improved resolution. A 1962 isolate from Tampa Bay, Florida, GHA-3 (FL-62A), was designated clade IIB by Kramer & Chandler (2001), but in the phylogenetic trees constructed using the expanded collection of sequences available for this study, this isolate consistently groups closer to clades IIA, IIC and IID than IIB, and should be considered in a clade of its own, designated IIG. Recent sequence data by Baillie and others (unpublished data available on GenBank) and confirmed by this study, suggest errors in the previously published sequences of MO-33 and GUA-69. Our MO-33 sequence is identical to BRA-68, and therefore falls into clade IIA. With this change, MO-37 also falls in this clade, resulting in the removal of the clade previously designated IIE. The Guatemalan isolate, GUA-69, was previously thought to be the only example of a recombinant of SLEV (Twiddy & Holmes, 2003), but recent sequencing has shown this strain to be a unique, non-recombined virus, most closely related to, but not identical to, TN-74. It therefore falls into clade IIB, removing clade IIF. In summary, lineage II previously contained six clades, IIA–IIF. With the addition of these isolates, this lineage now contains five clades: IIA, IIB, IIC, IID and IIG; clades IIE and IIF have been removed.

Twenty-five of the 28 isolates sequenced in this study were isolated in Texas, and all of those isolated from the Gulf Coast counties (Harris, Jefferson and Nueces County) fall into clade IIB, while those from El Paso County fall into clade VA. Clade IIB includes isolates from the 1980s and 1990s from the Gulf Coast region of Texas, TX-91A (Nueces County), TX-83 and TX-98 (both Harris County), a 1974 isolate from Tenessee (TN-74), and the resequenced GUA-69. In this clade, isolates TN-74 and GUA-69 are the most divergent from the Texas strains; sharing between 97.6 and 98.0 % identity with the other isolates, suggesting that this clade was introduced to Texas between the late 1970s and early 1980s, and has remained the dominant clade in the Gulf Coast region since that time. The Harris County isolates from 1998, 2001 and 2003, and the Jefferson County isolates from 2002 share the greatest identity (ranging from 99.1 to 99.9 %). Many of these nucleotide differences are non-coding changes, with the Jefferson County isolates having an identical amino acid sequence to the 1998 isolate from Harris County and many of the 2001 Harris County isolates (data not shown). The isolates from Nueces County are slightly more divergent, sharing between 98.1 and 98.6 % identity with the Harris and Jefferson County isolates, respectively. This was expected, given the geographical location of these counties, with Jefferson County being adjacent to Harris County, while Nueces County is more distant.

The two new isolates from El Paso County (western Texas) fall within clade VA with several temporally diverse isolates from Central and South America, and recent isolates from California. These El Paso isolates show greater identity to these isolates than any other isolates from Texas (99.7 and 100 % identity with the California isolates compared with only 91.8–92.4 % identity with the Gulf Coast Texas isolates). These data suggest that in this region of Texas, isolates have been introduced from California or South America rather than other parts of Texas, and originate from a different introduction to that of the Gulf Coast isolates. Sequencing of additional isolates from areas around the USA/Mexico border over the last 20 years would allow stronger conclusions to be made.

The results shown in this study, in particular those relating to the Texas Gulf Coast isolates, show that the same strain of SLEV circulates from year to year with little sequence divergence, suggesting that rather than being reintroduced every year, the virus survives the winter. The method of overwintering of SLEV in Texas has yet to be determined. A number of possible methods have been proposed in the past, depending on whether the virus remains local from year to year, or if the virus is reintroduced from tropical and subtropical areas each year. Isolates in the cooler, temperate regions of Texas, such as El Paso, share more in common, genetically, with Californian and South American isolates, suggesting that in this part of Texas, SLEV isolates are reintroduced from warmer regions, while in the case of the subtropical Gulf Coast region of Texas, where mosquito vectors may be active throughout the year, the genetic similarity of temporally diverse isolates implies overwintering rather than reintroduction, possibly by maintenance within the mosquito population during the colder months. Interestingly, a similar conclusion was drawn from a recent study of WNV over time in the Harris County area since its introduction in 2002, suggesting that the virus overwinters rather than annual reintroductions (Davis *et al.*, 2007a).

Like many other arboviruses that utilize an arthropod host in addition to a mammalian or avian host (Ciota *et al.*, 2007; Holmes, 2003; Jerzak *et al.*, 2005; Weaver & Barrett, 2004; Weaver, 2006; Woelk & Holmes, 2002), isolates of SLEV do not exhibit a high level of genetic diversity; indeed, the most genetically diverse isolates have only 10.1 % nucleotide divergence and strains within each lineage show less than 5.5 % nucleotide divergence (data not shown). Interestingly, this contrasts with the closely related viruses in the JEV complex, JEV and WNV, both of which show a higher level of divergence between strains [up to 22.6 % nucleotide and 11.2 % amino acid divergence between the most divergent JEV isolates (Uchil & Satchidanandam, 2001; Yamanaka *et al.*, 2006) and up to 24.6 % nucleotide and 11.8 % amino acid divergence between WNV isolates (Berthet *et al.*, 1997; Charrel *et al.*, 2003; Lanciotti *et al.*, 1999, 2002; Scherret *et al.*, 2001)]. In contrast, MVEV and SLEV show low levels of divergence [(10.1 % nucleotide and 2.4 % amino acid divergence for MVEV (Johansen *et al.*, 2007; Lobigs *et al.*, 1988) and 10 % nucleotide and 2.8 % amino acid divergence for SLEV)]. SLEV does not have an inherently lower mutation rate than WNV, indeed under certain conditions SLEV mutates at a faster rate, as observed when both viruses were serially passaged in C6/36 cells (Ciota *et al.*, 2007). These differences may relate to different selection pressures between the different viruses due to differing geographical locations and transmission cycles. In comparison to the geographical distribution of SLEV (confined to the New World only), WNV has a very large and diverse geographical distribution. Isolates are found in Africa, Europe, India, Australia and now the Americas. In general, outside of frequent gene exchange between Africa and Europe (Charrel *et al.*, 2003; Lanciotti *et al.*, 2002), and the introduction of WNV into North America from the Middle East (Lanciotti *et al.*, 1999), greater genetic diversity of WNV corresponds to a greater geographical range of isolates. In particular, WNV isolates from India or Australia are genetically distinct from other WNV isolates from Africa and Europe, but show little diversity within these geographical regions (Beasley *et al.*, 2002; Lanciotti *et al.*, 1999, 2002; Savage *et al.*, 1999; Scherret *et al.*, 2001). Indeed, isolates of WNV from the Gulf Coast region of Texas share similar levels of divergence as SLEV isolates from the same region (Davis *et al.*, 2007b). In contrast, the higher genetic diversity of JEV in comparison to other viruses may correspond to the different ecological cycle of this virus. Unlike most other viruses in the JEV complex, JEV utilizes pigs as an amplifying host in addition to birds (Halstead & Jacobson, 2003). All of the five genotypes of JEV circulate in Asia, where the human, bird and pig hosts all live in high densities and in close proximity to each other, allowing increased efficiency of infection and higher mutation rates (Gould *et al.*, 2003; Halstead & Jacobson, 2003). In contrast, MVEV has a limited geographical distribution, found in Australia and Papua New Guinea only (Johansen *et al.*, 2007; Lobigs *et al.*, 1988), where the vertebrate hosts are sparsely distributed. SLEV, found only in the Americas, has a limited geographical distribution compared with WNV, and unlike JEV, does not include pigs in its transmission cycle, utilizing mainly avian vertebrate hosts (Day, 2001). The amino acid changes observed in SLEV do not correspond to any known virulence determinants, and are likely the result of neutral mutations not affecting fitness or virulence rather than positive selection.

## Supplementary Material

[Supplementary table]

## Figures and Tables

**Table 1. t1:** Strains of SLEV sequenced in this study

**GenBank accession no.**	**Name**	**Designation**	**Year of isolation**	**Location**
EU306883	BFS508	CA-50	1950	CA
EU306884	BFN1324	CA-70B	1970	CA
EU306885	J7532	JAM-62	1962	Jamaica
EU306909	V4683	TX-91B	1991	Harris County, TX
EU306886	01V1933	TX-01A	2001	Harris County, TX
EU306887	01V1936	TX-01B	2001	Harris County, TX
EU306888	01V1937	TX-01C	2001	Harris County, TX
EU306889	01V2086	TX-01D	2001	Harris County, TX
EU306890	01V2088	TX-01E	2001	Harris County, TX
EU306891	01V2089	TX-01F	2001	Harris County, TX
EU306892	01V2211	TX-01G	2001	Harris County, TX
EU306893	01V2220	TX-01H	2001	Harris County, TX
EU306894	01V2231	TX-01I	2001	Harris County, TX
EU306895	01V2233	TX-01J	2001	Harris County, TX
EU306896	01V2892	TX-01K	2001	Harris County, TX
EU306897	01V2906	TX-01L	2001	Harris County, TX
EU306898	TDH1121	TX-02A	2002	Nueces County, TX
EU306899	TDH3178	TX-02B	2002	El Paso County, TX
EU306900	TDH3372	TX-02C	2002	Nueces County, TX
EU306901	TDH3438	TX-02D	2002	Jefferson County, TX
EU306902	TDH3439	TX-02E	2002	Jefferson County, TX
EU306903	TDH4074	TX-02F	2002	Nueces County, TX
EU306904	TDH4462	TX-02G	2002	El Paso County, TX
EU306905	TDH5307	TX-02H	2002	Nueces County, TX
EU306906	TDH6983	TX-02I	2002	Nueces County, TX
EU306907	TVP9042	TX-03A	2003	Harris County, TX
EU306908	TVP9041	TX-03B	2003	Harris County, TX
EU306910	LADERLE	UNK-66	1966	USA

**Table 2. t2:** Envelope protein amino acid changes specific to lineages

**Amino acid**	**Consensus**	**Lineage**												
		**IA**	**IB**	**IIA**	**IIB**	**IIC**	**IID**	**IIG**	**III**	**IV**	**VA**	**VB**	**VI**	**VII**
29	S	–	–	–	–	–	–	–	–	–	–	–	–	G
51	T	–	–	–	–	–	–	A	–	–	–	–	–	–
66	D	–	–	–	–	–	–	–	–	–	–	–	–	N
126	T	I	–	–	–	–	–	–	–	–	–	–	–	–
151	S	–	–	–	–	–	–	–	–	T	–	–	T	–
156	S	–	–	F/S	–	F/S	–	F	–	–	F/S	F/S	–	A
161	K	–	Q	–	–	–	–	–	–	–	–	–	–	–
176	F	Y	Y	–	–	–	–	–	–	–	–	–	–	–
201	Y	–	–	–	–	–	–	–	–	–	–	–	H	–
310	T	–	–	–	–	–	–	–	–	–	–	–	–	I
316	T	A	A	T/A	T/A	–	–	–	T/A	A	A	A	A	–
344	A	–	–	–	–	–	–	–	–	V/L	–	–	–	–
384	I	–	–	–	–	–	–	–	–	–	–	–	–	V
391	T	–	T/S	–	–	–	–	–	–	A	–	–	–	–
442	V	–	I	–	–	–	–	–	–	–	–	–	–	–
487	A	–	–	–	–	–	–	T	–	–	–	–	–	–
488	V	–	–	–	–	–	–	–	–	–	–	–	–	T
