# Supplementary material for: Genetic variation of St. Louis encephalitis virus
Source: J Gen Virol. 2008 Aug;89(Pt 8):1901–10. doi: 10.1099/vir.0.2008/000190-0 (PMC2696384; doi:10.1099/vir.0.2008/000190-0)
Supplement: [Supplementary table] [file supp_89_8_1901__index.html]

 Genetic variation of St. Louis encephalitis virus -- May et al. 89 (8): 1901 Data Supplement - Supplementary table -- Journal of General Virology

## 

### Genetic variation of St. Louis encephalitis virus, by F. J. May, L. Li, S. Zhang, H. Guzman, D. W. C. Beasley, R. B. Tesh, S. Higgs, P. Raj, R. Bueno, Y. Randle, L. Chandler and A. D. T. Barrett

*Journal of General Virology* vol. **89**, part 8, pp. 1901 - 1910

**Supplementary Table**  [PDF]  (74 KB)

  
  
