# Supplementary material for: Genetic variation of St. Louis encephalitis virus
Source: J Gen Virol. 2008 Aug;89(Pt 8):1901–10. doi: 10.1099/vir.0.2008/000190-0 (PMC2696384; doi:10.1099/vir.0.2008/000190-0)
Supplement: [Supplementary table] [file supp_89_8_1901__1.pdf]

Supplementary Table S1.

Lineage	GenBank accession no.	Strain	Designation	Year	Location
IA	EU306883	BFS508	CA-50	1950	CA
IA	AF205455	BFS1750	CA-53	1953	Kern Co., California
IA	AF205454	BFS4772	CA-63	1963	Kern Co., California
IA	AF205453	E22924	CA-70	1970	Kern Co., California
IA	EU306884	BFN1324	CA-70B	1970	CA
IB	AF205457	IV-824	CA-78	1978	Imperial Co., California
IB	AF205458	SOUE 135	CA-85	1985	Los Angeles, California
IB	AF205456	COAV750	CA-98	1998	Riverside Co., California
IB	AF205497	72V4749	CO-72	1972	Washington Co., CO
IB	AF205494	72V1165	NM-72	1972	Chaves Co., NM
IB	AF205496	TD6-4G	TX-66A	1966	Dallas, TX
IB	AF205493	P17797	TX-66B	1966	Dallas, TX
IB	AF205495	68V1587	TX-68	1968	Hale Co., TX
IB	AF205491	PV7-3389	TX-87	1987	El Paso Co., TX
IB	AF205492	PV0-620	TX-89	1989	Dallas, TX
IIA	AF205472	SpAn9398	BRA-68	1968	Sao Paulo, Brazil
IIA	AF205460	69M-1143	FL-69A	1969	Polk Co., Florida
IIA	AF205459	L695121.05	FL-69B	1969	South Florida
IIA	EU306885	J7532	JAM-62	1962	Jamaica
IIA	AF205506	904	KY-56	1965	Calvert City, KY
IIA	EF158070	Parton	MO-33	1933	St Louis, MO
IIA	AF205508	Hubbard	MO-37	1937	St Louis, MO
IIA	AY289618	MSI-7	MS-75	1975	Mississippi
IIA	AF205510	TexU1193	TX-54	1954	TX
IIA	AF205512	Texas1955	TX-55A	1955	Rio Grande Valley, TX

IIA	AF205511	TexM6	TX-55B	1955	TX
IIA	AF205507	Tex16017	TX-59	1959	TX
IIA	EU306910	LADERLE	UNK-66	1966	USA
IIB	EF158051	GMO-94	GUA-69	1969	Guatemala
IIB	EF158057	78A28	GUA-U	?	Guatemala
IIB	AF205501	TNM4-711k	TN-74	1974	Memphis, TN
IIB	EU306886	01V1933	TX-01A	2001	Harris Co, TX
IIB	EU306887	01V1936	TX-01B	2001	Harris Co, TX
IIB	EU306888	01V1937	TX-01C	2001	Harris Co, TX
IIB	EU306889	01V2086	TX-01D	2001	Harris Co, TX
IIB	EU306890	01V2088	TX-01E	2001	Harris Co, TX
IIB	EU306891	01V2089	TX-01F	2001	Harris Co, TX
IIB	EU306892	01V2211	TX-01G	2001	Harris Co, TX
IIB	EU306893	01V2220	TX-01H	2001	Harris Co, TX
IIB	EU306894	01V2231	TX-01I	2001	Harris Co, TX
IIB	EU306895	01V2233	TX-01 J	2001	Harris Co, TX
IIB	EU306896	01V2892	TX-01K	2001	Harris Co, TX
IIB	EU306897	01V2906	TX-01L	2001	Harris Co, TX
IIB	EU306898	TDH1121	TX-02A	2002	Nueces Co., TX
IIB	EU306900	TDH3372	TX-02C	2002	Nueces Co., TX
IIB	EU306901	TDH3438	TX-02D	2002	Jefferson Co, TX
IIB	EU306902	TDH3439	TX-02E	2002	Jefferson Co, TX
IIB	EU306903	TDH4074	TX-02F	2002	Nueces Co., TX
IIB	EU306905	TDH5307	TX-02H	2002	Nueces Co., TX
IIB	EU306906	TDH6983	TX-02I	2002	Nueces Co., TX
IIB	EU306907	TVP9042	TX-03A	2003	Harris Co, TX
IIB	EU306908	TVP9041	TX-03B	2003	Harris Co, TX
IIB	AF205498	83V4953	TX-83	1983	Harris Co., TX

IIB	AF205499	PV1-2419	TX-91A	1991	Nueces Co., TX
IIB	EU306909	V4683	TX-91B	1991	Harris Co., TX
IIB	AF205500	98V3181	TX-98	1998	Harris Co., TX
IIB	EF158052	V2380-42	UNK-U	?	USA
IIC	AF205463	Imp917	CA-88B	1988	Imperial Co., California
IIC	AF205465	Kern217	CA-89	1989	Kern Co., California
IIC	AF205461	Imp1311	CA-91A	1991	Imperial Co., California
IIC	AF205464	Chlv374	CA-91C	1991	Riverside Co., California
IIC	AF205462	Chlv587	CA-92A	1992	Riverside Co., California
IIC	AY135518	COAV 608	CA-U		Coachella Valley, CA
IIC	AF205467	FL79-411	FL-79	1979	Florida
IIC	AF205502	7526PG-3	MD-75	1975	Maryland
IIC	AF205504	Fort Washington	MD-77A	1977	Maryland
IIC	AF205503	VP34	MD-77B	1977	Prince Georges City, MD
IIC	AF205466	75v14868	TN-75	1975	Memphis, TN
IID	AF205469	TBH-28	FL-62B	1962	Tampa Bay, FL
IID	AF205468	P15	FL-62C	1962	Tampa Bay, FL
IID	AF205470	65V-310	MEX-65	1965	Mexico
IID	AF205471	PanAn 902604	PAN-73A	1973	Panama
IIG	AF205505	GHA-3	FL-62A	1962	Tampa Bay, FL
III	DQ385450	CbaAr4006	ARG-05A	2005	Cordoba, Argentina
III	DQ385451	CbaAr4005	ARG-05B	2005	Cordoba, Argentina
III	AF205490	79V2533	ARG-79	1979	Santa Fe, Argentina
III	DQ022950	SPH253157	BRA-04	2004	Sao Paulo, Brazil
IV	AF205476	PanAr 902745	PAN-73B	1973	Panama
IV	AF205475	GML902612	PAN-73C	1973	Panama
IV	AF205489	GML902981	PAN-77A	1977	Panama

IV	AF205477	GML902984	PAN-77B	1977	Panama
IV	AF205488	GML900968	PAN-U	?	Panama
VA	AF205481	78V6507	ARG-78	1978	Santa Fe, Argentina
VA	AF205478	BeAr242587	BRA-73A	1973	Belem, Brazil
VA	AF205482	BeAn246407	BRA-73B	1973	Belem, Brazil
VA	AF205480	BeAn248398	BRA-U	?	Belem, Brazil
VA	EF158067	BeAn247377	BRA-U	?	Brazil
VA	AY135511	COAV 332	CA-00A	2000	Coachella Valley, CA
VA	AY135514	COAV 405	CA-00B	2000	Coachella Valley, CA
VA	AY135516	COAV 444	CA-00C	2000	Coachella Valley, CA
VA	AY135517	COAV 470	CA-00D	2000	Coachella Valley, CA
VA	AY135512	COAV 353	CA-01A	2001	Coachella Valley, CA
VA	AY135513	COAV 363	CA-01B	2001	Coachella Valley, CA
VA	AY135515	COAV 411	CA-01C	2001	Coachella Valley, CA
VA	AY135510	COAV 297	CA-98B	1998	Coachella Valley, CA
VA	AF205479	75D90	PER-75	1975	Peru
VA	AF205486	TR9464	TRIN-55	1955	Trinidad
VA	EU306899	TDH3178	TX-02C	2002	El Paso, TX
VA	EU306904	TDH4462	TX-02H	2002	El Paso, TX
VB	AF205485	BeAr23379	BRA-60	1960	Belem, Brazil
VB	AF205484	BeH203235	BRA-71	1971	Belem, Brazil
VB	AF205483	BeAn246262	BRA-72	1972	Belem, Brazil
VI	AF205487	GML903797	PAN-83	1983	Panama
VII	AF205473	CorAn9124	ARG-66	1966	Cordoba, Argentina
VII	NC_007580	Argentine66	ARG-66B	1966	Argentina
VII	AF205474	CorAn9275	ARG-67	1967	Cordoba, Argentina

May, F. J., Li, L., Zhang, S., Guzman, H., Beasley, D. W. C., Tesh, R. B., Higgs, S., Raj, P., Bueno, R., Randle, Y., Chandler, L. and Barrett, A. D. T. (2008). Genetic variation of St. Louis encephalitis virus. *J Gen Virol* **89**, 1901–1910.
